# Patient and public involvement (PPI) in UK surgical trials: a survey and focus groups with stakeholders to identify practices, views, and experiences

**DOI:** 10.1186/s13063-019-3183-0

**Published:** 2019-02-11

**Authors:** Joanna C. Crocker, Keira Pratt-Boyden, Jenny Hislop, Sian Rees, Louise Locock, Sophie Olszowski, Alan Chant, Shaun Treweek, Jonathan A. Cook, Kerry Woolfall, Nicola Farrar, Jennifer Bostock, Richard Bulbulia

**Affiliations:** 1 Health Experiences Research Group, Nuffield Department of Primary Care Health Sciences, University of Oxford, Oxford, UK; 2 NIHR Oxford Biomedical Research Centre, Oxford, UK; 3 MRC ConDuCT-II (Collaboration and innovation for Difficult and Complex randomised controlled Trials In Invasive procedures) Hub for Trials Methodology Research, Bristol Medical School, Bristol, UK; 4 School of Anthropology and Conservation, University of Kent, Canterbury, UK; 5 Formerly Health Experiences Research Group, Nuffield Department of Primary Care Health Sciences, University of Oxford, Oxford, UK; 6 Oxford Academic Health Science Network, Oxford, UK; 7 Health Services Research Unit, University of Aberdeen, Aberdeen, UK; 8 Formerly NIHR Oxford Biomedical Research Centre and Unit, Oxford, UK; 9 Patient Partner, Berkshire, UK; 10 Surgical Intervention Trials Unit, Nuffield Department of Orthopaedics, Rheumatology and Musculoskeletal Sciences, University of Oxford, Oxford, UK; 11 Institute of Psychology, Health and Society, University of Liverpool, Liverpool, UK; 12 MRC North West Hub for Trials Methodology Research, Liverpool, UK; 13 Population Health Sciences, Bristol Medical School, University of Bristol, Bristol, UK; 14 Formerly Surgical Intervention Trials Unit, Nuffield Department of Surgical Sciences, University of Oxford, Oxford, UK; 15 Lay partner, Kent, UK; 16 MRC CTSU (Clinical Trial Service Unit) Hub for Trials Methodology Research, Nuffield Department of Population Health, University of Oxford, Oxford, UK; 17 MRC Population Health Unit, Nuffield Department of Population Health, University of Oxford, Oxford, UK; 18 Cheltenham General Hospital, Gloucestershire Hospitals NHS Foundation Trust, Cheltenham, UK

**Keywords:** Patient and public involvement (PPI), Surgery, Survey, Focus group, Clinical trial

## Abstract

**Background and aims:**

Historically, patient and public involvement (PPI) in the design and conduct of surgical trials has been absent or minimal, but it is now routinely recommended and even required by some research funders. We aimed to identify and describe current PPI practice in surgical trials in the United Kingdom, and to explore the views and experiences of surgical trial staff and patient or public contributors in relation to these practices. This was part of a larger study to inform development of a robust PPI intervention aimed at improving recruitment and retention in surgical trials.

**Methods:**

Our study had two stages: 1) an online survey to identify current PPI practice in active UK-led, adult surgical trials; and 2) focus groups and interviews with key stakeholders (surgical trial investigators, administrators, and patient or public contributors) to explore their views and experiences of PPI.

**Results:**

Of 129 eligible surgical trial teams identified, 71 (55%) took part in the survey. In addition, 54 stakeholders subsequently took part in focus groups or interviews. Sixty-five (92%) survey respondents reported some kind of PPI, most commonly at the design and dissemination stages and in oversight or advisory roles. The single most common PPI activity was developing participant information sheets (72%). Participants reported mixed practice and views on a variety of issues including the involvement of patients versus lay members of the public, recruitment methods, use of role descriptions and payment for the time of PPI contributors. They suggested some solutions, including the use of written role descriptions and databases of potential PPI contributors to aid recruitment.

**Conclusions:**

UK surgical trials involve patients and members of the public in a variety of different ways, most commonly at the beginning and end of the trial lifecycle and in oversight or advisory roles. These are not without challenges and there remain uncertainties about who best to involve, why, and how. Future research should aim to address these issues.

**Electronic supplementary material:**

The online version of this article (10.1186/s13063-019-3183-0) contains supplementary material, which is available to authorized users.

## Lay summary

Patient and public involvement (or ‘PPI’ for short) means researchers working with patients and members of the public in all or any parts of research. This could include choosing the research topic, prioritising from a list of research ideas, designing, planning and doing research, and communicating the findings of research to different groups of people. PPI is becoming increasingly common in health research, including clinical trials, which are a common way of testing new medicines and other treatments.

This paper describes a survey we did to find out what kind of PPI is happening in 71 surgical trials in the United Kingdom. (By ‘surgical trials’ we mean clinical trials which test a new type of surgery, or some other treatment in patients who are having or have had surgery.) We also talked to 54 people interested in surgical trials, to find out what they think about PPI, and how it could be improved.

Almost all the surgical trials in our survey (92%) were doing some kind of PPI. This was most common at the beginning of the trial (helping to design it) and at the end of the trial (helping to communicate the findings to different groups of people). It was more common for patients and members of the public to be involved as independent advisers than as members of the trial team. The most common thing they were asked to do was help design the patient information sheet. This is the information given to patients when they are invited to take part in a clinical trial.

The people we spoke to had experienced some challenges with PPI in surgical trials and had different opinions about how PPI should be done. These included whether to involve patients with the health condition being studied or lay members of the public, how to find patients and members of the public to be involved, and whether or not to pay them for their time. They also suggested some ways PPI could be improved, such as having written ‘job’ descriptions and building up a database of people interested in being involved.

Some questions remain about who best to involve and how, and we hope future research will be able to answer these.

## Background

Patient and public involvement (PPI) in research has been defined as ‘research being carried out “with” or “by” members of the public [including patients] rather than “to”, “about” or “for” them’ [1]. This includes, for example, working with research funders to prioritise research, offering advice as members of a project steering group, commenting on and developing research materials, and undertaking interviews with research participants [1]. Clinical trials in the UK have experienced a recent surge in PPI activity, partly because the National Institute for Health Research (NIHR) now expects active PPI in the research it funds [2]. There is also a new research agenda for PPI in clinical trials [3] and resources to facilitate the planning, reporting, and evaluation of PPI [4–6].

Despite an increasing focus on the importance of PPI in trials, in a cohort investigation of NIHR- funded trials conducted between 2006 and 2010 only 25% of surgical intervention trials detailed PPI in the outline grant application, compared with 75% of other clinical trials (*p* = 0.01) [7]. Similarly, in a systematic review of PPI in surgical trials in 2014, PPI was rarely reported in publications [8], although an absence of reporting does not necessarily mean an absence of PPI. In this study, we sought to: 1) identify and describe current PPI practice in surgical trials; and 2) explore the views and experiences of surgical trial staff and PPI contributors (involved patients and members of the public) in relation to these practices, including their advantages and disadvantages.

This study comprises the first and second stages of a larger project funded by the MRC Network of Hubs for Trials Methodology Research to develop a PPI intervention aimed at enhancing recruitment and retention in surgical trials (PIRRIST) [9]. In order to develop an effective intervention, we needed to first identify baseline PPI activity among UK surgical trials.

## Methods

### Stage 1: survey

The primary objective of this survey was to ascertain current PPI practice in UK surgical trials. We also explored respondents’ attitudes towards PPI.

#### Survey design

Informed by a framework developed by Oliver et al. [10] and the findings of qualitative research led by a co-author (LL) on the experiences of PPI contributors in medical and health research [11–13], we agreed that the following themes would be included in the survey:

- Rationale for including or not including PPI
- Role(s) of PPI contributors
- Number of PPI contributors involved
- Activities undertaken by PPI contributors
- Mode(s) of interaction between PPI contributors and researchers
- PPI contributor characteristics (e.g. person with condition under study, lay person, etc.)
- Methods used to recruit PPI contributors
- Presence or absence of written documentation outlining PPI roles
- Support/guidance/resources used to inform PPI
- Funding for PPI
- Respondent’s beliefs about PPI
- Lessons learned from respondents’ experiences of PPI

Where possible, we used or adapted items from existing PPI questionnaires (from outside surgical research) to formulate our initial survey questions [7, 14–19]. We then piloted the survey iteratively with a convenience sample of 13 trial staff, including nine trial managers and four clinical investigators. Cognitive debriefing, namely the ‘think aloud’ technique, was used with each pilot participant (either face-to-face or by telephone) to identify difficulties in interpreting or responding to questions. Piloting continued until no further changes were required. The final survey (Additional file 1) took participants 10–15 min to complete. It mainly consisted of closed questions, with optional free-text comment boxes on every page.

As a starting point, we used the definition of PPI provided by INVOLVE [1] (a national, government-funded advisory group for PPI in health and social care research) and added further clarification during piloting. The final definition of PPI used in the survey is shown in Table 1.

**Table 1:** Definition of patient and public involvement (PPI) used in this survey

By ‘PPI’ we mean researchers consulting with or working alongside members of the public, patients, service users, and/or carers in all or any part(s) of the research process, including the choice of research topic, design, planning, conduct, and/or dissemination of research. In this survey we refer to these people as ‘PPI contributors’. PPI contributors may be, for example, grant co-applicants, members of the Trial Steering Committee or Trial Management Group, members of a patient or lay advisory panel, or participants in a trial-specific consultation exercise such as a focus group, survey, or interviews. Consultation exercises may or may not use formal research methods. By ‘PPI’, we do not mean researchers recruiting people to be participants in the trial, or researchers disseminating information about the trial to patients or the public.	

#### Identification of eligible trials

Trials eligible for the survey were active, UK-led trials of surgical interventions or other interventions in adult surgical patients. ‘Active’ meant that they were in set-up (i.e. funded and pending regulatory approvals), open to recruitment, or closed to recruitment and in follow-up.

Eligible trials were identified in three ways:

1. A search of the UK Clinical Research Network (CRN) online database of portfolio studies [20] listed under the ‘surgery’ specialty. The UK CRN portfolio of studies consists of high-quality research studies that are eligible for consideration for support (in developing, setting up, and delivering high-quality clinical research) from the NIHR-funded Clinical Research Network in England. At the time of this survey, the database was publicly available via the UK CRN website.
2. The Royal College of Surgeons portfolio of surgical trials in England.
3. Knowledge of eligible trials through personal connections.

#### Survey delivery

We identified 129 eligible trials and sent a personal email invitation to the primary contact listed for each eligible trial. In the invitation, potential participants were offered a £10 high street shopping voucher or academic book voucher as a ‘thank you’ for their time; this was sent by post on completion of the survey. Personal email reminders were sent to non-responders at 2 and 4 weeks after the initial invitation. The survey was open for a total of 12 weeks between September and November 2015. Our participant information sheet was based on a user-tested template developed by Knapp et al. [21], and we used the Bristol Online Surveys tool [22] to deliver the survey. The identity and contact information of respondents were requested at the end of the survey to enable us to keep in touch with respondents and deliver thank-you vouchers, but this was optional and respondents could complete the survey anonymously if preferred. However, we did ask for the trial name or acronym at the beginning of the survey to check eligibility and carry out a response bias analysis. Respondents did not have to complete the survey in one sitting (there was a ‘finish later’ option), but their responses were not submitted until completion of the whole survey.

In the hope of increasing the response rate to personal invitations, prior to and during the survey period we carried out several awareness-raising activities among surgical research staff: 1) seminars to staff audiences at six academic surgical research centres in the UK (Oxford, Aberdeen, Bristol, Birmingham, London, Leicester); 2) an online blog published on the NIHR Oxford Biomedical Research Centre website; 3) promotional flyers distributed to delegates at the UK Trial Managers Network annual meeting; 4) a national webinar hosted by the MRC Network of Hubs for Trials Methodology Research—Trial Conduct Working Group; 5) promoting the study via Twitter; and 6) informing personal contacts.

#### Survey analysis

We exported the survey data into IBM SPSS Statistics 22 and generated simple statistical summaries of the closed form responses to each survey item. Free-text qualitative data were grouped thematically and used to aid interpretation of the quantitative data where relevant. Data were checked for inconsistencies and respondents contacted for clarification where necessary and if they had given their permission to be re-contacted for this purpose. We explored whether trials funded by the NIHR (fully or partially) and with later recruitment start dates would be more likely to have PPI in the funding application. For both factors, a difference in the percentage of trials with PPI in the funding application was calculated with the 95% confidence interval (CI) using Newcombe’s model 10 using the rdci command in Stata version 15.

#### Assessment of response bias

We hypothesised that our sample of respondents would be biased towards those with experience of PPI in surgical trials (since the topic would be of greater interest to people already doing PPI). In order to test this hypothesis and estimate the degree of response bias, we obtained relevant data from the National Research Ethics Service (NRES) through a Freedom of Information request for each trial invited to take part in the survey. These data consisted of the responses (including free-text comments) to question A14–1 of the NRES application form: “In which aspects of the research process have you actively involved, or will you involve, patients, service users, and/or their carers, or members of the public?” A researcher (JCC) used this information to code each trial as ‘PPI’ or ‘No PPI’. A difference in the percentage of trials with PPI between responders and non-responders was calculated using the same method as noted above.

### Stage 2: focus groups

In the context of the wider PIRRIST project, the primary objectives of this applied qualitative research were to explore: 1) views and experiences (especially challenges) of recruitment, retention, and PPI in surgical trials; 2) views about the impact of PPI on recruitment and retention of surgical trial participants; and 3) possible ideas for the PIRRIST intervention. This paper focuses on the first of these objectives in relation to PPI.

Eligible participants were UK-based current surgical trial staff (any role, including PPI coordinator) and PPI contributors with combined experience of PPI, clinical trials (any intervention) and surgery or surgical research. Eligible trial staff were identified from stage 1 survey respondents, open adverts (via email and Twitter) distributed to and cascaded by regional and national PPI and surgical networks/groups, and our own professional contacts. Prior to conducting the focus groups, we also published a promotional article in the *Bulletin of the Royal College of Surgeons of England* [23].

Interested potential participants were asked to indicate which of four sites (Oxford, Aberdeen, Birmingham, or Bristol) they would prefer to attend. Potential participants at each site were then asked to complete a Doodle poll showing their availability. Travel bookings and overnight accommodation were offered to ensure that geography was not a barrier. We aimed to recruit eight participants per focus group with a diverse range of roles and experiences; staff focus groups had to include at least one trial manager, principal investigator, and research nurse. Dates which best fulfilled these criteria were chosen. Potential participants who were unable to join a focus group, as well as the focus group participants themselves, were invited to submit (additional) comments in writing if they wished. To maximise participation by PPI contributors, those unable to attend a focus group were offered an alternative one-to-one interview in person or by telephone. Focus group and interview participants were offered a £20 high street shopping voucher or book voucher as a ‘thank you’ for taking part.

Focus groups were facilitated by a non-clinical member of the research team (JCC), who used a semi-structured topic guide covering the following: experiences of PPI; experiences of participant recruitment and retention; participants’ views of the impact of PPI on participant recruitment and retention (including how this happens); and ideas for the PIRRIST intervention. The focus groups were audio-recorded, and an observer (KPB) took notes to aid transcription. The audio-recordings were transcribed verbatim, checked, and anonymised before undergoing thematic analysis [24]. The first full transcript was coded deductively by three researchers independently (JCC, KPB, JH) against the pre-specific topics of interest: views and experiences of PPI; suggestions for improving PPI; participant recruitment; participant retention; impact of PPI on recruitment; impact of PPI on retention; other impacts of PPI; ideas for PPI intervention. The coding was discussed, agreed, and transferred to NVivo. The coding reports (coded text within each of the pre-specified topics) were then coded inductively by the same three researchers, and a preliminary thematic framework was agreed. This framework was then applied to subsequent transcripts independently by two researchers (JCC and KPB), who regularly discussed, agreed, and refined the framework.

### Combining stage 1 and stage 2 findings

Themes which emerged from analysis of the focus groups were mapped against the themes covered in the survey to identify areas of overlap. In this paper, we present the findings under cross-cutting themes (i.e. those for which we have information regarding both the frequency of practice and people’s views and experiences of the practice). Participant ID numbers are provided where direct quotations are used, with ‘SR’ indicating a stage 1 survey respondent, ‘PS’ indicating a stage 2 staff participant, and ‘PP’ a stage 2 patient or lay participant. The notation ‘[…]’ is used to indicate where verbatim text has been excluded from the quotation. In some cases, details have been removed and replaced with broad descriptors (e.g. ‘[medical condition]’) to ensure anonymity.

### PPI in this study

The idea for the PIRRIST study emerged from meetings with an advisory panel for JCC’s research fellowship, which was funded by the NIHR Oxford Biomedical Research Centre to research PPI impact assessment. The advisory panel included two patient advisers (including author AC), who were involved in the group to ensure that the research was relevant to, and informed by the perspectives of, patients and members of the public. They were chosen because of their long-term experience of PPI and interest in assessing its impact. The decision to undertake the PIRRIST study (and an accompanying systematic review [25]) was in part due to our patient advisers’ desire to measure the impact of PPI, particularly on patient recruitment to clinical trials. Whilst the study was underway, one patient adviser (MO) retired and a third (RH) joined the group. These patient partners provided input at six advisory group meetings and email correspondence between meetings, and one (AC) was co-applicant on the project grant and continued to be a member of the study team throughout. A second lay partner (JB) joined the study team once funding had been secured. As well as helping to conceive of the PIRRIST study, the patient partners and advisers helped to design the overall study and its patient-facing materials (online survey, focus group topic guide, information sheets, invitations, and adverts), promote the study to wider patient/PPI groups, and interpret the findings. As a team, we believe that PPI is worth doing but that it should be evaluated to improve practice and maximise value.

## Results

### Stage 1 survey respondents

Of 129 eligible trials that were identified, and the primary contacts invited to take part, 71 (55.0%) participated in the survey. We were unable to source NRES PPI data for 29 (22.5%) of the invited trials because the question about PPI had not been implemented at that time or NRES was unable to retrieve the data. For the 99 trials with NRES PPI data available, 49/56 (87.5%) survey responders reported PPI or plans for PPI in the NRES application form, compared with 33/43 (76.7%) non-responders, with a difference in percentage of trials with PPI input of 11% (95% CI −4 to 26%).

At the time of survey completion, 7 (9.9%) participating trials were in setup, 46 (64.8%) were open to recruitment, and 18 (25.4%) were closed to recruitment and in follow-up. Recruitment start dates ranged from July 2004 to June 2017 (median July 2013). Of these trials, 54 (76.1%) were funded by the National Institute for Health Research, 6 (8.5%) by another public funder, 16 (22.5%) by a charity, and 3 (4.2%) by industry. (These categories are not mutually exclusive; 8 trials (11.1%) had multiple funder types.) Cancer, cardiovascular disease, and musculoskeletal conditions were the most common clinical specialties (16 trials; 22.5% each).

The survey respondents included 40 (56.3%) trial managers, 17 (23.9%) chief investigators, 6 (8.5%) co-investigators, 6 (8.5%) other trial staff, and 2 (2.8%) PPI coordinators. They represented a wide range of views on PPI (Table 2), although the vast majority of respondents agreed or strongly agreed that PPI is morally/ethically the right thing to do (91.5%) and that PPI can make a positive difference to surgical trials (87.3%).

**Table 2:** Survey respondent beliefs about patient and public involvement (PPI) (*n* = 71)

	Number of trials (%)
PPI is morally/ethically the right thing to do	
• Strongly agree	29 (40.8%)
• Agree	36 (50.7%)
• Undecided	2 (2.8%)
• Disagree	2 (2.8%)
• Strongly disagree	2 (2.8%)
PPI can make a positive difference to surgical trials	
• Strongly agree	24 (33.8%)
• Agree	38 (53.5%)
• Undecided	6 (8.5%)
• Disagree	1 (1.4%)
• Strongly disagree	2 (2.8%)
PPI can improve the recruitment of participants to surgical trials	
• Strongly agree	19 (26.8%)
• Agree	37 (52.1%)
• Undecided	13 (18.3%)
• Disagree	1 (1.4%)
• Strongly disagree	2 (2.8%)
PPI can improve the retention of participants in surgical trials	
• Strongly agree	16 (22.5%)
• Agree	32 (45.1%)
• Undecided	21 (29.6%)
• Disagree	1 (1.4%)
• Strongly disagree	1 (1.4%)

### Stage 2 focus group and interview participants

A total of 54 people (including 31 surgical trial staff, 21 PPI contributors, and 2 PPI coordinators) took part in stage 2 between January and June 2016. We conducted six focus groups: four with surgical trial staff (at the universities of Oxford, Aberdeen, Birmingham, and Bristol) and two with PPI contributors (both at the public library of Birmingham). In addition to the focus groups, we carried out seven one-to-one interviews with PPI contributors (two face-to-face and five by telephone) and received 11 written contributions.

### Frequency of PPI in UK surgical trials

Sixty-five (91.5%) surveyed trials reported that there was, or had been, PPI in the trial according to our definition. The most commonly cited reasons for including PPI in the trial were that it was considered morally/ethically the right thing to do, that it was believed to result in better research, and that it was required by the funder(s) (Table 3). Five of the six trials which did not have any PPI gave at least one reason: PPI was not a requirement when the trial was set up (*n* = 4); PPI was unlikely to improve the trial (*n* = 3); and the trial team had tried but failed to identify PPI contributors (*n* = 1).

**Table 3:** Reasons for including patient and public involvement (PPI) in the trial (*n* = 65)

	Number of trials (%)
Considered morally or ethically the right thing to do	47 (66.2%)
Believed to result in better research	46 (64.8%)
Required by funder(s)	44 (62.0%)
To improve recruitment of participants to this trial	40 (56.3%)
To improve retention of participants in this trial	28 (39.4%)
Institutional policy	19 (26.8%)
PPI contributor(s) offered their services	2 (2.8%)
Do not know	1 (1.4%)

### Who were the PPI contributors?

**Fig. 1:**
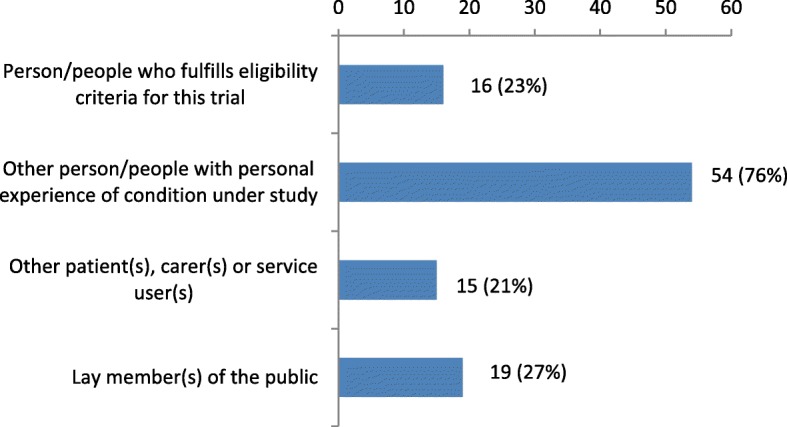
PPI contributors (*n* = 71 trials)

Of the 71 surveyed trials, three-quarters had PPI contributors with personal experience of the condition under study but who did not fulfil the eligibility criteria for that trial, while a minority of trials had other patient(s), carer(s) or service user(s), lay members of the public, or patients who fulfilled the eligibility criteria for the trial (Fig. 1).

Stage 2 participants discussed the merits of having PPI contributors with experience of the medical condition under study versus lay members of the public with no such experience. In focus group 4 (trial staff), lay members of the public were described positively as ‘reminding the professionals about the patient’ (PS25), ‘almost like a mediator’ between researchers and patients (PS24), and ‘a neutral kind of person’ (PS24). It was suggested that a lay person might feel more able to challenge the research team than a patient:

‘I wonder if a lay person as well, wouldn’t have that…maybe that sort of feeling of power imbalance as much as a patient would, with the other—the academics and the professionals—they might just be, ‘Well, I have no sort of experience of this and I have no reason to not say anything to upset this person because I'm never going to be seen by them, or I'm never going be in that sort of community,’ so maybe they would feel more able to speak up in some ways.’ (PS24, PPI coordinator, focus group 4)

Lay people were also seen as more able to commit to long-term trials than patients with serious conditions: ‘…if you’ve got palliative patients they can’t sort of sit on steering group and the trials that go on for years.’ (PS25, PPI coordinator, focus group 4).

However, lay people were sometimes perceived to have an alternative agenda that could steer the focus away from patients. One surgical investigator (PS28) gave an example of PPI in setting a research agenda for a life-threatening condition. Most contributors present at the meeting were members of the public and were interested in a related, more common condition which ‘doesn’t kill you’. This distortion ‘defined the whole day for us’ and the smaller group of patients present at the meeting felt frustrated that their life-threatening condition was not prioritised more in discussions. The investigator concluded that ‘if you’re dealing with lay people you have to understand their agenda—why are they there?’

Some participants felt PPI contributors should have experience of the condition under study, or even be typical of the target population:

‘I can talk about what has happened to me [but] if you suddenly said, right, will you go on a trial for somebody who’s got earache or asthma, I wouldn’t know, have a clue.’ (PP56, PPI contributor, interview)

‘I think it’s very important to recruit [PPI contributors] who have experience of what the programme is about. […] There’s no, no point in asking a nurse what the exhaust content of an internal combustion engine…’ (PP53, PPI contributor, interview)

‘We've got two patients who sit on our trial steering committee, but they are professional patients—so one's an ex-GP and one's an ex-university dean. […] But I am very conscious that perhaps in some sense, although they are real patients, they’ve both actually, you know, had their surgical procedure that we're doing the trial to look at, which is invaluable, but they're not exactly, you know Joe Bloggs off the street you know, they are professionals.’ (PS23, trial manager, focus group 4)

However, patients or carers were sometimes felt to have vested interests which could be problematic for the trial team, especially if their role was unclear:

‘He [involved carer] wasn’t very clear about what his role was I don’t think, and he kind of turned… he gave the impression that he'd like to turn our trial—all our trials, or all trials even—into trials into a particular sub-section of the disease that his daughter had, and ended up doing all sorts of research on his own, sending emails to the chief investigator at all sorts of times. "Ooh have you seen this, have you read this?" and so on. And yeah, he could have been either better selected or better informed.’ (PS13, trial manager, focus group 2)

‘I think you can sometimes get the people that attend who've got a slightly alternative agenda and definitely hope that it will give them better healthcare or give them access and I think it's very hard to sort of keep it… keep them back a little bit and not let them completely take over the group…’ (PS06, research nurse, focus group 1)

One patient on a trial management team spoke of the need to involve the trial participants themselves, and the difficulty of doing this:

‘I think that for people like myself actually working on behalf of the patients in a trial management team for instance, then we need access to the patients somehow. In a way you feel a little bit distanced. […] I have my own personal journey but I can't use that particularly in the trial management teams that I'm associated with. So, I like to think I can get a hold of patients who are currently involved and actually are in the actual trial participating, and that’s something that’s a bit frustrating that we can't…it's not easy to do that.’ (PP11, PPI contributor, focus group 6)

### How were the PPI contributors recruited?

The most common way in which PPI contributors were recruited was through asking people the trial team already knew, particularly patients or former patients of a clinician on the team (Table 4). Nearly half of the trials approached an established group, service, or organisation, while very few used open adverts. In only two cases did PPI contributors approach the trial team.

**Table 4:** How was/were the patient and public involvement (PPI) contributor(s) recruited? (*n* = 65 trials with some kind of PPI)

	Number of trials (%)
Asked person/people already known to member(s) of the trial team	40 (61.5)
• Patient(s) or former patient(s) of a clinician on the team	32 (49.2)
• PPI contributor(s) from a previous study	12 (18.5)
• Participant(s) from a previous study	4 (6.2)
• Acquaintance(s), friend(s) or relative(s)	4 (6.2)
• Participant(s) from this trial	2 (3.1)
• Other	2 (3.1)
• Do not know	1 (1.5)
Approached an established group, service, or organisation	33 (50.8)
• A patient group or voluntary organisation	19 (29.2)
• An established PPI group in my research centre/institution	9 (13.8)
• Research Design Service (RDS)	2 (3.1)
• Clinical Research Network (CRN)	2 (3.1)
• Other	4 (6.2)
Open invitation/advert (e.g. newspaper, website, poster)	5 (7.7)
PPI contributor(s) approached the trial team	2 (3.1)
Other	1 (1.5)
Do not know	4 (6.2)

Inviting patients already linked to a clinician on the team was considered by stage 2 participants to be an effective way to recruit PPI contributors, but potentially limiting the impact PPI could have, because it could result in ‘yes men’:

‘I advised the consultant to put—the PI [Principal Investigator]—to put, you know, “consultant” on an individual letter to patients from the database and I am convinced that that did the trick. That people had had that personal invitation to a lunch and a mini-seminar really.’ (PP50, PPI contributor, interview)

‘I think from my perspective, in my trial, the PPI was identified by the CI [Chief Investigator], both of them, because I've got two—one's a PMG [Project Management Group] member and the other one's a TSC [Trial Steering Committee] member—and I would say that cronyism is potentially a downside to that, because it's obvious that the CI talks to them, out-with those meetings, and they just agree with everything that happens in the meeting, and with the [funder], are specifically asked for their involvement in the reports and they just say they're very happy with how everybody…everybody's working really well, and that’s all they say. […] So, I'm not sure that they're necessarily the best people for my trial because they're yes men and they aren’t necessarily providing bad news for the team to consider.’ (PS20, trial manager, focus group 3)

The recruitment of PPI contributors via established patient or public groups, services, or organisations was seen as providing several benefits including patients motivated to contribute, and access to a wider group of patients and communication platforms:

‘So, I would cling to that resource [patient support groups], cos I think with all this because I think that if they're motivated enough to go along to one of those groups, they’ll be motivated enough probably to help the PPI...’ (PS04, trial manager, focus group 1)

‘…we've used a patient charity that we have connections with to raise awareness of the trial, and it's quite a rare disease with not many people who suffer from it, so that’s been quite useful. […] We've had sort of consultation with groups of patients that we've reached through the charity that we probably wouldn’t have had if we hadn’t been advertising them.’ (PS13, trial manager, focus group 2)

However, some participants feared that PPI contributors from patient organisations might be too ‘professionalised’ for the role:

‘We've had a variety of PPI people work on the trials, and we've had one who was very involved with a patient charity, and at times it feels like we're dealing with a sort of professional PPI person rather than somebody who is actually still a patient.’ (PS13, trial manager, focus group 2)

‘Well I was just wondering what the group thought about patient groups and representatives of patient groups, because you can speak to, say, the head of a patient group, who in theory speaks for their…you know their entire community, and that person might be a professional patient and might be, you know very well educated and have gone back to represent the views of people, you know all sorts of people. Do you guys feel it's reasonable to talk to the head of that patient group on the assumption that they do represent their members, or is that not an appropriate person to recruit?’ (PS28, investigator, focus group 4)

Paradoxically, professional skills and an ability to think beyond their own patient experience were considered useful or even necessary:

‘If your background is in management, if your background is in academia or whatever, those skills can be transferred to a multi-disciplinary panel where you are on a same wavelength as academics or whatever. So, the skills come from not only your patient experience which is experience engagement, but involvement goes a bit deeper.’ (PP04, PPI contributor, focus group 5)

‘We did some focus groups, well, it was a study actually, at [hospital name], of what patients thought of the [medical specialty] questionnaires and a lot of them couldn’t get what we were saying: Is this question sensible? Does it relate to your life? And they just answered the question and they couldn’t reflect on whether it was a sensible question or not.’ (PP50, PPI contributor, interview)

Open advertisements were seen as good practice and more inclusive, but could be challenging due to the additional time required:

Female participant (unidentified): ‘I think a lot of the time you’ve got to identify your PPI people before you’ve even got the funding up and running do you think?’

Female participant (unidentified): ‘Yeah it's a very, very early stage in that I don’t really know if you’ve got time to advertise fully…’ (Focus group 3)

However, open advertisements could still feel frustratingly exclusive, as voiced by one patient participant who applied for a PPI opportunity advertised as ‘first come, first served’:

‘…on first come, first served, it’s quite—the difficulties of that is, one, you have to be on email, two, you have to look at your email at the right time and they did say they expected it to be well over-subscribed before the final deadline…’ (PP56, PPI contributor, interview)

Several participants mentioned that a database or ‘pool’ of interested people would be a useful recruitment resource:

‘Having a database of patients who are willing to be involved in the PPI process and the specialities in which they have user experience would be a really useful tool to have in each research department.’ (PS50, research nurse, email contribution)

‘The only way to do that is for each [university] department to have their standing PPI group. Of patients, as recent as possible.’ (PP50, PPI contributor, interview)

### Where did PPI feature in the research process?

Of the 71 surveyed trials, most had PPI in research design (82%) and the dissemination of findings (59%), while a minority of trials had PPI in undertaking the research (24%) and in the analysis and/or interpretation of results (31%). By far the most common PPI activity was developing participant information materials such as information sheets and consent forms (72% of trials). However, PPI limited to developing patient information sheets was generally regarded as tokenistic:

‘It’s very easy for it [PPI] to be tokenistic, and I think if you’ve just got like one individual who occasionally comes along to a trial team and is just shown an information leaflet and stuff, and things like that, then you don’t properly engage them there...’ (PS12, trial manager, focus group 2)

‘I've definitely seen a shift in the level of involvement of PPI. It has really, really changed. At the start it did almost feel like it was…I probably shouldn’t say this, but it did almost feel like it was a tick box exercise, it was something that the funders asked for, so you had to have a patient representative on your TMG [Trial Management Group], possibly on your TSC [Trial Steering Committee], and have them feed into your PIS [patient information sheet] and consent forms.’ (PS12, trial manager, focus group 2)

There was general agreement that PPI should begin earlier in the trial process in order to maximise the potential for positive impact:

‘Somebody was doing a [research study] on a [device] to encourage [medical condition] patients to visualise the injured hand. It was set around a task in the kitchen of making a cup of tea. This entire programme was designed, and somebody spent ages making it all pretty, to encourage a [medical condition] patient to be able to move both hands to make a cup of tea. One of the patients just turned round and said, "Everyone can make a cup of tea one handed, try doing something like buttering toast," and the designer was just [clicks fingers] deflated like that. I thought, 'You should have asked patients a year ago.' So yes, that made a massive difference to the future research because they essentially told him, 'Go back and start again.' It proved that you really need to put patients in much earlier, so it was very valuable.’ (PS31, trial manager, focus group 4)

‘Ideally, involve, involving the patients and people who have experienced the condition very early on at the research question stage. Because I’ve seen so many times where PPI has been brought in at the late stage, when it’s all been decided and it’s very hard to comment on something at that stage. There’s too much at stake. Too much would have to change and so really right from deciding the research question.’ (PP50, PPI contributor, interview—in response to being asked what PPI means to them)

PPI in choosing the research topic or question was reported by fewer than one quarter of trials, while PPI in developing the funding application and data collection tools were each reported by approximately half of trials (Table 5). PPI plans seemed to be fluid, with some free-text comments indicating that PPI might be added at later stages of the trial, particularly at dissemination.

**Table 5:** Patient and public involvement (PPI) in stages of the research process (*n* = 71)

	Number of trials (%)
Research design	58 (81.7%)
• Research topic or question	16 (22.5%)
• Funding application	33 (46.5%)
• Intervention design	21 (29.6%)
• Participant information materials (e.g. information sheets, consent forms, recruitment adverts)	51 (71.8%)
• Data collection tools (e.g. questionnaires, interview schedules)	36 (50.7%)
• Recruitment methods	29 (40.8%)
• Retention methods	19 (26.8%)
• Do not know	1 (1.4%)
• Other^a^	2 (2.8%)
Undertaking the research	17 (23.9%)
• Promoting the trial to encourage recruitment	9 (12.7%)
• Identifying or screening potential participants	8 (11.3%)
• Taking consent from participants	1 (1.4%%)
• Collecting research data	2 (2.8%%)
• Do not know	2 (2.8%)
• Other^b^	1 (1.4%)
Analysis and/or interpretation of results	22 (31.0%)
• Analysing research data	3 (4.2%)
• Interpreting data or results	16 (22.5%)
• Do not know	1 (1.4%)
• Other^c^	3 (4.2%)
Dissemination of findings	42 (59.2%)
• Writing or reviewing research reports	11 (15.5%)
• Writing or reviewing lay summaries	32 (45.1%)
• Presenting the findings at a research conference	6 (8.5%)
• Presenting the findings to a lay audience	24 (33.8%)
• Suggesting routes/platforms for dissemination	30 (42.3%)
• Do not know	3 (4.2%)
• Other	0 (0.0%)
None of the above	0 (0.0%)
Other^d^	2 (2.8%)

^a^Outcome measures (*n* = 2)

^b^Developing a video/DVD to aid informed consent (*n* = 1)

^c^Reviewing interim reports at Trial Steering Committee (TSC) meetings (*n* = 1); discussing results with PPI group (*n* = 2)

^d^General oversight or management of the research (*n* = 2)

Trials funded by the NIHR were not more likely to have PPI in the funding application (48% vs. 41%; 7% difference, 95% CI −19 to 31%), nor were trials with a recruitment start date on or after the median of 1st July 2013 (54% vs. 38%; 16% difference, 95% CI −7 to 37%).

### PPI roles within surgical trials

The majority of surveyed trials had at least one PPI contributor on the Trial Steering Committee (72%), while fewer had a PPI contributor as grant co-applicant (35%) and/or member of the Trial Management Group (35%) (Table 6). Over 60% of trials also consulted PPI contributors out-with these roles; methods included focus group or group discussion (*n* = 25), interviews (*n* = 21), email consultation (*n* = 11), survey (*n* = 6), online group discussion or forum (*n* = 1), and other informal methods. Other roles mentioned by survey respondents in free-text included PPI membership of the independent Data Monitoring Committee (*n* = 1) and PPI in investigator training and interviewing for the trial physician post (*n* = 1).

**Table 6:** Patient and public involvement (PPI) contributor roles within surgical trials (*n* = 71)

	Number of trials (%)
Co-applicant(s) on grant	25 (35.2%)
• 1 co-applicant	23 (32.4%)
• 2 co-applicants	2 (2.8%)
Formal member(s) of Trial Management Group or equivalent study team	25 (35.2%)
• 1 member	15 (21.1%)
• 2 members	8 (11.3%)
• 3 members	2 (2.8%)
Member(s) of Trial Steering Committee	51 (71.8%)^a^
• 1 member	31 (43.7%)
• 2 members	16 (22.5%)
• 3 or more members	2 (2.8%)
Consultee(s)	45 (63.4%)^b^
• 1–5 consultees	11 (15.5%)
• 6–10 consultees	10 (14.1%)
• 11–20 consultees	9 (12.7%)
• More than 20 consultees	5 (7.0%)

^a^Two trials did not have a Trial Steering Committee at the time of survey completion

^b^Includes focus group or group discussion (*n* = 25), interviews (*n* = 21), email consultation (*n* = 11), survey (*n* = 6), online group discussion or forum (*n* = 1), and other informal methods (*n* = 7). In the case of 10 trials, all or part of this consultation was a formal research project (requiring ethics approval and informed consent from participants)

Despite PPI co-applicants being present in over one-third of surveyed trials, there was evidence that this was sometimes tokenistic, as illustrated by survey respondent SR50, a trial manager: ‘…they [PPI co-applicants] were listed on the grant but I do not think they had much input to the design.’

An interesting finding was the use of a two-tier model of PPI by several trials, in which a smaller number of PPI contributors were closely and regularly involved with the trial team, linked to a larger group of patients who were consulted intermittently. This model was seen as beneficial because it resulted in better patient engagement due to greater relevance and the opportunity for social networking:

‘I've been using patient panels rather than individual patients, because my experience of using individual patients within surgical trials has not been good because of non-attendance and non-engagement. So, I've had much more success in creating panels of patients who have one or two representatives of those panels who'll come to steering groups to represent the group as a whole.’ (PS19, principal investigator, focus group 3)

‘If you do it in a bigger forum where at least there's more than one PPI person, if you do it in a group, then I think they’ll feel like they can engage better and they’ve got somebody else there with them that they can connect with, and they don’t feel like they're there by themselves. […] I think that’s the best way of really getting patients talking to each other, exchanging ideas and really feeling like they're involved in the study.’ (PS12, trial manager, focus group 2)

‘We found it's worked really well to have it separate actually, and you can just focus on the things that need talking about with them, rather than I suppose them having to sit through an entire meeting where maybe only certain bits of it might be relevant for them. […] I think it started off working well and then it didn’t and they couldn’t attend the meetings and it just wasn’t working, so we took a different approach and so far it seems to be working well. […] In addition to how it helps the trial, I think patients really value coming along to a meeting of just patients and just all sharing their stories actually.’ (PS24, PPI coordinator, focus group 4)

However, one participant warned of the difficulty initiating this kind of arrangement, because ‘the Trial Manager is often not involved in the trial when this sort of group needs to be set up’ (SR69, trial manager, survey respondent).

### Use of PPI role descriptions

When asked the question ‘Has/have the PPI contributor(s) been given a written document outlining their role(s) in this specific trial?’, only 10 (15.4%) surveyed trials with PPI responded ‘yes—all PPI contributors’ and 17 (26.2%) responded ‘yes—some PPI contributors’. Despite these low frequencies, role descriptions were viewed by both surgical trial staff and PPI contributors as useful tools for recruiting suitable and diverse PPI contributors, and even necessary from an ethics point of view:

‘There’s something about actually understanding what it is that you’re wanting from your PPI, and actually having a role description and making sure that what you’re doing is matching people against those role descriptions. So, that might be about how much time is going to be involved in it. It might be about the cultural aspects of it. It might be about we need somebody who’s going to be able to go out there and sell it, whatever it is, but you need to be able to encapsulate that but you also need to be able to make some matching against it and not—what happens I think quite a lot in research is that there’s just like anybody who actually shows any sign of interest, it’s like ‘Well, we’ll take them now!’ [laughs], and that lowers the quality of PPI, so consequently it lowers the inequality of the research generally.’ (PP02, principal investigator, focus group 1)

‘But one of the other things in [Borough] as well that was pointed out to me, was that they felt, with hindsight, that you needed to make sure that the PPI representatives had a very accurate description of what their roles and responsibilities were at the outset, so that they knew before they actually consented to take part really, about what they were taking on… (PS09, research nurse, focus group 2).

### Payment for PPI contributors’ time and expenses

With regard to funding for PPI, 35 (53.8%) trials had specific funding for their PPI, which was usually included in the research grant (33 trials; 50.8%). Almost all surveyed trials with PPI reimbursed PPI contributors for any travel and/or out of pocket expenses related to their involvement: 48 (73.8%) always and 10 (15.4%) sometimes (free-text responses indicated that PPI contributors sometimes declined or failed to claim expenses). However, PPI contributors were usually not paid for their time related to involvement (e.g. with vouchers, honoraria, or direct payment), with 38 (58.5%) trials reporting that this never happened. Surgical trial staff and PPI contributors had mixed views about payment; some saw it as essential recognition of work done and part of equalising the relationship with researchers, as illustrated by the below conversation among PPI contributors in focus group 5, and others as ‘interfering with the taxman’ (PP01, PPI contributor) or potentially attracting people for the wrong reasons:

PP03: ‘…I don’t classify it as a payment or a fee. What I see it as [is] recognition of time sacrifices. So, if I’m emailing yourself with anybody else I might say, ‘In addition to my travelling expenses, would there be any recognition of [inaudible] like two or three hours travelling to a venue, two or three hours going back?’ And the things I have to do for my sister whilst I’m away, who’s going to do that? Do I have to pay somebody else? Those tasks don’t get done by themselves, so it’s just…it’s not so much a payment as in, going to work and getting paid a hundred and fifty or two hundred pounds a day, it’s a recognition of the times I’ve…it’s a small amount, that’s all we ask for but, a recognition.’

PP04: ‘And you’re seriously considered as a partner then or something…a serious partner.’

PP02: ‘It’s something about the recognition within the group that you’re dealing with…you’ve got everybody else who sits round the table with you—health economists, statisticians, clinicians or what have you, who are being paid, and therefore I think that the way that they treat you is almost as an amateur because you’re seen as an amateur, and the more that we can do to professionalise it almost for that…’ (focus group 5)

‘… I think PPI is taking off in a big way, I’m not sure if it’s for the right reasons because now there’s always a payment with it, isn’t there? But on saying that if someone’s got a job and they have to take time off work they need to be paid. If they’ve got carers, they need to be paid.’ (PP04, PPI contributor, focus group 5)

‘We had one [PPI rep] who was what we consider a “professional patient” and they charged for their time—quite a lot of money. The other PPI rep did not do this and I feel we got more from the unpaid rep as they were doing it out of the goodness of wanting to help rather than trying to make money from it and contributing in a tokenistic manner.’ (SR54, trial manager, survey)

## Discussion

### Main findings

Our survey findings suggest that PPI has started to become routine practice for UK surgical trials, with over 90% of surveyed trials reporting some kind of PPI. Patients and members of the public were reportedly involved in a variety of different ways, most commonly at the design and dissemination stages (relative to the trial conduct and data analysis stages) and in oversight or advisory roles (relative to partnership or management roles). The single most common PPI activity was developing participant information sheets (72% of surveyed trials), but there was evidence that this was sometimes tokenistic, and general agreement that PPI should be started earlier in surgical trial design. A two-tier model of PPI, in which a small number of PPI contributors are closely involved with the trial team and linked to a larger group of patients, was seen as beneficial because it resulted in a better representation and patient engagement than the involvement of one or two PPI contributors alone. This is consistent with the finding of a realist evaluation that a similar ‘outreach’ model of PPI, in which lay representatives are linked to broader communities, was an effective and efficient model of PPI in clinical research [26].

Almost none of the surveyed trials included participants from the trial as PPI contributors. However, one PPI focus group participant suggested that input from trial participants would be a useful form of PPI, and expressed frustration that participants are difficult to access for this purpose. Vale et al. recently recommended that PPI guidance be updated to routinely consider including participants as part of wider PPI plans [27].

PPI contributors usually had personal experience of the condition under study (either as patients or carers), and this experiential knowledge was viewed by some as being a crucial attribute of PPI. Nevertheless, public contributors without such experience could bring advantages for the trial team too, such as impartiality and a greater ability to commit to the long-term trial in some medical contexts. This is consistent with previous research which identified the ‘expert in lived experience’ as only one of several potential roles embodied by PPI contributors [12].

PPI contributors were recruited most commonly via a clinician on the team; other means had proved difficult for some participants, and a database or pool of interested people was suggested as a potentially useful resource for surgical trialists. Formal role descriptions for PPI contributors were not commonly used, but were viewed as a potentially useful tool for recruitment of suitable candidates. Reimbursement of PPI contributors’ expenses was common practice, but payment for time was less common, and participants had mixed feelings about the appropriateness of payment.

Many of the challenges and views identified in this study are not unique to surgical trials and have been widely reported for PPI in clinical trials and health research more broadly, including tokenism, lack of clarity around PPI roles, difficulty recruiting and retaining PPI contributors, and issues around payment and funding [11, 16, 28–31]. We did not identify any unique, stand-alone issues that would apply to surgical trials but not to other types of trials, although the relative importance of some of the shared issues and uncertainties may differ (a question beyond the scope of this study).

It is worth noting that some of the discourses identified in this study have been or could be critiqued. For example, the common view that some PPI contributors are too ‘professionalised’ for the role has been criticised as oversimplistic^34^. Some training and/or expertise is often helpful, and the degree and type required (or not) will vary according to the specific role. The cronyism of clinical investigators choosing favourite patients as PPI contributors was criticised but is consistent with the manner in which many other research team members (e.g. the statistician or health economist) are routinely chosen. It perhaps reflects the view that part of a PPI contributor’s role is to be a ‘challenging outsider’^11^ , and that this may not be possible if investigators have a tokenistic attitude towards PPI (deliberately choosing people they anticipate will be compliant) or if there is an inherent power imbalance, such as when clinical investigators choose their own current patients. Whilst involving current patients may be unwise, we would argue that choosing former patients with whom clinical investigators already have a positive relationship is not necessarily a bad thing, and in fact may be beneficial, since successful PPI appears to depend on establishing and maintaining good interpersonal relationships [26, 32].

### Strengths and limitations

Our survey yielded a high response rate relative to surveys of PPI practice in health research more broadly [16] and was not subject to significant response bias. However, our findings may be somewhat historical, since PPI practice is changing rapidly [19] and many of the trials in our sample began several years ago. PPI is likely to be even more common and more embedded now than it was when we conducted our survey.

Another limitation is that this was not a true mixed-methods study, but rather a quantitative study followed by a qualitative study. While there was some overlap between the survey respondents and focus group participants, we deliberately sought a wider range of perspectives for the focus groups (including, for example, PPI contributors and research nurses); therefore, some of the surgical trials mentioned by focus group participants may not have been included in the survey and vice versa. Nevertheless, we believe that the qualitative dataset helps shed some light on the ‘real-life’ experiences and views surrounding the PPI practices identified in the survey.

None of the focus group or interview participants questioned whether PPI should be performed in surgical trials, nor were we able to recruit any of the few survey respondents with negative beliefs about PPI, suggesting that the findings may not include the full range of views on this topic. Finally, we struggled to recruit eligible industry-sponsored trials to this study, succeeding with only one of eight identified. Our findings are therefore based almost exclusively on trials sponsored by academic institutions and the National Health Service and may not be generalisable to commercial trials.

### Implications for surgical trials and future research

The findings of this study will inform the development of a robust PPI intervention aimed at improving recruitment and retention in surgical trials (PIRRIST), which enhances rather than duplicates baseline PPI practice. The findings may also help surgical trialists planning PPI for new trials; the survey findings provide a benchmark against which they could assess their plans (how do their PPI plans compare with their peers?), while the focus group findings highlight some of the advantages and disadvantages of different PPI practices. Further research exploring how best to involve the public and patients in the design stage of surgical trials, including the benefits and challenges of involving trial participants as PPI contributors, would be valuable.

## Conclusions

PPI has started to become routine practice in academic- and NHS-sponsored UK-based surgical trials, most commonly involving one or two patients in advisory or oversight roles such as membership of the Trial Steering Committee. However, there is potential for, and signs of a shift towards, much greater and earlier involvement.

## Additional files

Additional file 1: Survey content. (PDF 392 kb)

Additional file 2: Anonymised survey dataset. (XLSX 63 kb)

## Abbreviations

CI: Confidence interval
CRN: Clinical Research Network
NRES: National Research Ethics Service
PIRRIST: Developing a Patient and public Involvement intervention to enhance Recruitment and Retention In Surgical Trials
PPI: Patient and public involvement
