# Supplementary material for: Patient and public involvement (PPI) in UK surgical trials: a survey and focus groups with stakeholders to identify practices, views, and experiences
Source: Trials. 2019 Feb 11;20:119. doi: 10.1186/s13063-019-3183-0 (PMC6371592; doi:10.1186/s13063-019-3183-0)
Supplement: Supplementary file 1 — Survey content. (PDF 392 kb) [file 13063_2019_3183_MOESM1_ESM.pdf]

PPI in surgical trials - version 1 (21 August 2015)

Welcome

Thank you very much for agreeing to take part in this survey of patient and public involvement (PPI) in surgical trials; by doing so you will help us to develop a PPI intervention that is as useful as possible to surgical trialists.

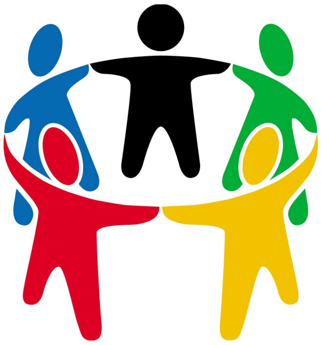

There are **optional comments boxes** at the bottom of each page of the survey; you can use these for anything you wish to tell us, or not use them at all.

At the end of the survey you will have the opportunity to choose a **£10 "thank you" voucher** (high street shopping voucher or Blackwell's book voucher) and to receive a copy of the results of this study if you wish.

Please allow around **10 minutes** to complete the survey.

If you have any questions, need help completing this survey, or would prefer a paper version, please don't hesitate to contact our team by emailing pirrist@phc.ox.ac.uk or calling **01865 617837.**

Thank you very much for your help.

Oxford Biomedical Research Centre

NHS National Institute for Health Research

UNIVERSITY OF OXFORD

UNIVERSITY OF ABERDEEN

UNIVERSITY OF LIVERPOOL

MRC Hubs for Trials Methodology Research

Consent

For the purposes of informed consent, please confirm the following:

1. I have read the study **information sheet [hyperlink to PDF]** (version 1, dated 21 August 2015), had the opportunity to ask questions and received satisfactory answers.

2. I understand that this project has been reviewed by, and received ethics clearance through, the University of Oxford Central University Research Ethics Committee.

3. I understand that my participation is voluntary and that I am free to withdraw myself or my data at any time, without giving any reason, and without any negative consequences.

4. I understand who will have access to personal data provided.

5. I understand how personal data will be stored and what will happen to the data at the end of the project.

6. I understand what will happen to the results of the study.

7. I understand how to raise concerns or make a complaint.

I have read and understood the above statements and agree to take part in this survey ✱ *Required*

Trial details

Please enter the **short title** or **acronym** of your trial:

[ ]

At what stage is this trial currently?

- ○ In set up
- ○ Open to recruitment
- ○ Closed to recruitment and in follow-up
- ○ Completed
- ○ Other

What is your role in the trial?

- ☐ Trial Manager or Trial Co-ordinator
- ☐ Chief Investigator
- ☐ Co-Investigator
- ☐ Other

If you selected Other, please specify:

[ ]

Comments: *Optional*

More info

PPI in your trial

You will now be asked some questions about patient and public involvement (PPI) in this trial. By 'PPI' we mean researchers **consulting with** or **working alongside** members of the **public**, **patients**, **service users** and/or **carers** in all or any part(s) of the research process, including the choice of research topic, design, planning, conduct and/or dissemination of research. In this survey we refer to these people as **'PPI contributors'**.

PPI contributors may be, for example: grant co-applicants, members of the Trial Steering Committee or Trial Management Group, members of a patient or lay advisory panel, or participants in a trial-specific consultation exercise such as a focus group, survey or interviews. Consultation exercises may or may not use formal research methods.

By 'PPI', we do **not** mean researchers recruiting people to be participants in the trial, or researchers disseminating information about the trial to patients or the public.

Please contact the research team if you have any queries about this definition which might affect your responses in this survey (telephone 01865 617837 or email pirrist@phc.ox.ac.uk).

Is there, or has there been, any PPI in this trial according to the definition above?

- Yes
- No
- Don't know

Comments: *Optional*

More info

No PPI - details

What was the reason for not including PPI (according to our definition) in your trial? *Please tick all that apply.*

- ☐ It was not a requirement when this trial was set up
- ☐ It is unlikely to improve the trial
- ☐ It is too expensive
- ☐ It is too time consuming
- ☐ Don’t know
- ☐ Other

If you selected Other, please specify:

We acknowledge that there is no single, universally agreed definition of PPI in health research. If there is anything you have been doing, or plan to do, that you or the trial team consider to be PPI, please describe it here: *Optional*

Comments: *Optional*

Co-applicants

We will now ask you a series of questions about PPI in this trial including:

- Grant co-applicants
- Trial Management Group or equivalent trial team
- Trial Steering Committee
- Consultations (e.g. surveys, interviews, focus groups, online forums)
- Any other types of PPI

Are any PPI contributors included as **co-applicants** on the grant for this trial?

- ◯ Yes
- ◯ No
- ◯ Don’t know

If yes, **how many**? *Please select from drop-down list.*

Comments: *Optional*

Trial Management Group (TMG)

Are any PPI contributors formal members of your **Trial Management Group (TMG)** or equivalent study team?

More info

- Yes
- No
- Don’t know

If yes, **how many**? *Please select from drop-down list.*

Are any of these PPI contributors **also** co-applicants?

- Yes - all of them are co-applicant(s)
- Yes - some of them are co-applicant(s)
- No
- Don't know

Comments: *Optional*

Trial Steering Committee

Are any PPI contributors formal members of your **Trial Steering Committee (TSC)**?

More info

- Yes
- No
- Don’t know
- N/A - there is no TSC

If yes, **how many**? *Please select from drop-down list.*

Comments: *Optional*

Consultations

Apart from any PPI you have already told us about, have you consulted, or will you consult, **any other** patients, service users, carers or members of the public about any aspects of this trial?

- ○ Yes
- ○ No
- ○ Don’t know

If yes, **how many**? *Please select from drop-down list.*

Which of the following **methods** of consultation have been, or will be, used with this person or these people? *Please tick all that apply.*

- ☐ Online or paper survey(s)
- ☐ Face-to-face or telephone interview(s)
- ☐ Face-to-face focus group(s) or group discussion(s)
- ☐ Email consultation(s)
- ☐ Online group discussion(s) or forum(s)
- ☐ Other

If you selected Other, please specify:

Is/was all or part of this **consultation** a formal research project (requiring ethics committee approval and informed consent from participants)?

- Yes
- No
- Don’t know

Comments: *Optional*

Other types of PPI

Have you included, or will you include, PPI in your trial in any other way? (i.e. **not** as co-applicants, members of the Trial Management Group or trial team, members of the Trial Steering Committee, or consultants)

- Yes
- No
- Don't know

If yes, please describe in as much detail as possible:

Comments: *Optional*

PPI in research design, conduct, analysis & dissemination

In which aspects of the research process have you actively involved, or will you involve, patients, service users, and/or their carers, or members of the public?

- ☐ Research design
- ☐ Undertaking the research (e.g. identifying or recruiting participants, collecting data)
- ☐ Analysis and/or interpretation of results
- ☐ Dissemination of findings
- ☐ None of the above
- ☐ Other

If you selected Other, please specify:

Which aspects of **research design**?

- ☐ Research topic or question
- ☐ Funding application
- ☐ Intervention design
- ☐ Participant information materials (e.g. information sheets, consent forms, recruitment adverts)
- ☐ Data collection tools (e.g. questionnaires, interview schedules)
- ☐ Recruitment methods
- ☐ Retention methods
- ☐ Don't know
- ☐ Other

If you selected Other, please specify:

Which aspects of **undertaking the research**? *Please tick all that apply.*

- ☐ Promoting the trial to encourage recruitment
- ☐ Identifying or screening potential participants
- ☐ Taking consent from participants
- ☐ Collecting research data
- ☐ Don’t know
- ☐ Other

If you selected Other, please specify:

Which aspects of **analysis** and **interpretation**? *Please tick all that apply.*

- ☐ Analysing research data
- ☐ Interpreting data or results
- ☐ Don’t know
- ☐ Other

If you selected Other, please specify:

Which aspects of **dissemination**? *Please tick all that apply.*

- ☐ Writing or reviewing research reports
- ☐ Writing or reviewing lay summaries
- ☐ Presenting the findings at a research conference
- ☐ Presenting the findings to a lay audience
- ☐ Suggesting routes/platforms for dissemination
- ☐ Don’t know
- ☐ Other

If you selected Other, please specify:

Comments: *Optional*

PPI input

In which of the following way(s) have the PPI contributor(s) provided input, or will they provide input? *Please tick all that apply.*

- ☐ Face-to-face meeting(s)
- ☐ Teleconference(s)
- ☐ Telephone call(s) with member of research team
- ☐ Email(s)
- ☐ Social media messages (e.g. Facebook, Twitter)
- ☐ Don't know
- ☐ Other

If you selected Other, please specify:

If they provide input via face-to-face meetings, who **chairs** these meetings? *Please tick all that apply.*

- ☐ A member of trial staff
- ☐ A patient or lay chair
- ☐ An external facilitator/chair
- ☐ Don't know
- ☐ Other

If you selected Other, please specify:

If they provide input via teleconferences, who **chairs** these teleconferences?
Please tick all that apply.

- ☐ A member of trial staff
- ☐ A patient or lay chair
- ☐ An external facilitator/chair
- ☐ Don’t know
- ☐ Other

If you selected Other, please specify:

Comments: *Optional*

PPI setup

Who is/are the PPI contributor(s) you have told us about? *Please tick all that apply.*

- ☐ Person/people who fulfill(s) the eligibility criteria for this trial
- ☐ Other person/people with personal experience of the condition under study
- ☐ Other patient(s), carer(s) or service user(s)
- ☐ Lay member(s) of the public
- ☐ Don’t know
- ☐ Other

If you selected 'Other patient(s), carer(s) or service user(s)', please specify:

If you selected Other, please specify:

How was/were the PPI contributor(s) recruited? *Please tick all that apply.*

- ☐ Open invitation/advert (e.g. newspaper, website, poster)
- ☐ Asked person/people already known to member(s) of the trial team
- ☐ Approached an established group, service or organisation
- ☐ PPI contributor(s) approached the trial team
- ☐ Don’t know
- ☐ Other

If you selected Other, please specify:

If you selected 'Asked person/people already known to member(s) of trial team', please specify who:

- ☐ Patient(s) or former patient(s) of a clinician on the team
- ☐ PPI contributor(s) from a previous study
- ☐ Participant(s) from a previous study
- ☐ Participant(s) from this trial
- ☐ Acquaintance(s), friend(s) or relative(s)
- ☐ Don’t know
- ☐ Other

If you selected Other, please specify:

If you selected 'Approached an established group, service or organisation', please specify type(s):

- ☐ An established PPI group in my research centre / institution
- ☐ A patient group or voluntary organisation
- ☐ Research Design Service (RDS)
- ☐ Clinical Research Network (CRN)
- ☐ Don’t know

- [ ] Other

If you selected Other, please specify:

Has/have the PPI contributor(s) been given a written document outlining their role(s) in this specific trial?

- ( ) Yes - all PPI contributors
- ( ) Yes - some PPI contributors
- ( ) No
- ( ) Don’t know

Comments: *Optional*

Support for PPI

Has the PPI in this trial been informed by any of the following guidance or resources? *Please tick all that apply.*

- ☐ PPI policy or written guidance developed by my research centre or institution
- ☐ Advice from a PPI expert within my research centre or institution
- ☐ INVOLVE guidance documents and/or website
- ☐ Formal training in PPI for at least one member of trial staff
- ☐ Research Design Service
- ☐ None
- ☐ Don’t know
- ☐ Other

If you selected Other, please specify:

Are/were the PPI contributors reimbursed for any **travel and/or out of pocket expenses** related to their involvement?

- ○ Yes - always
- ○ Yes - sometimes
- ○ No
- ○ Don't know
- ○ N/A - no expenses were incurred by PPI contributors

Are/were the PPI contributors paid for their **time** related to involvement? (e.g. with vouchers, honoraria or direct payment)

- ◯ Yes - always
- ◯ Yes - sometimes
- ◯ No
- ◯ Don't know

Is there, or has there been, any specific **funding** for PPI in this trial?

- ◯ Yes
- ◯ No
- ◯ Don’t know

If yes, who provided this funding?

- ☐ It was part of the research grant
- ☐ My research centre / institution
- ☐ Research Design Service
- ☐ Don’t know
- ☐ Other

If you selected Other, please specify:

Comments: *Optional*

Reason(s) for including PPI in this trial

Why was PPI included in this trial? *Please tick all that apply.*

- ☐ Required by funder(s)
- ☐ Institutional policy
- ☐ Considered morally or ethically the right thing to do
- ☐ Believed to result in better research
- ☐ To improve recruitment of participants to this trial
- ☐ To improve retention of participants in this trial
- ☐ PPI contributor(s) offered their services
- ☐ Don’t know
- ☐ Other

If you selected Other, please specify:

Comments: *Optional*

Looking ahead

Based on your experience of PPI in this trial, would you do anything differently next time?

- Yes
- No
- Don't know

If **yes**, please explain:

Comments: *Optional*

Your beliefs about PPI

How much do you disagree or agree with the following statements?

	Strongly disagree	Disagree	Undecided	Agree	Strongly agree
PPI is morally/ethically the right thing to do	☐	☐	☐	☐	☐
PPI can make a positive difference to surgical trials	☐	☐	☐	☐	☐
PPI can improve the recruitment of participants to surgical trials	☐	☐	☐	☐	☐
PPI can improve the retention of participants in surgical trials	☐	☐	☐	☐	☐

Comments: *Optional*

Contact details

May we contact you if we need to clarify any of your responses to this survey?

- Yes
- No

As part of this study we would like to analyse written documents that are being used in PPI (e.g. institutional policy documents, role descriptions, templates). If you have indicated that you use documents like this, would you potentially be happy to share them with us?

- Yes
- No
- N/A - no written documents used

We are planning further phases of the research project to help develop a PPI intervention that is useful and acceptable (including focus groups, a second online survey and a consensus workshop). May we contact you with information about other phases of the project?

- Yes
- No

We would also like to invite PPI contributors who have been involved in surgical trials to take part in further phases of the project. Would you potentially be happy to pass on information about this opportunity to your PPI contributors?

- Yes

- No
- N/A - there is no PPI in this trial

Would you like us to let you know when the results of this project are available?

- Yes
- No

Which type of voucher would you like to receive as a “thank you” for completing this survey?

- £10 One4All voucher (accepted at over 20,000 leading high street stores)
- £10 Blackwell’s book voucher (can be redeemed online or in store)
- I would prefer not to receive a voucher

Please enter your **email address** so we know who you are and can act on your requests above:

More info

Please enter your **contact details** below so we can send you your voucher and/or contact you in the future (if you have given permission above)

More info

Full name (including title):	
Address line 1:	
Address line 2:	
Address line 3:	
City/Town:	
Postcode:	
Telephone number:	

Comments: *Optional*

Final comments (optional)

If there are any further comments you would like to make about PPI, this survey or our research project, please type in the box below:

Please click 'Finish' to complete this survey and allow us to see your responses. If you exit without clicking 'Finish', we will not receive any of your responses or any information about your identity.

If you have any queries please contact our research team (email pirrist@phc.ox.ac.uk or call 01865 617837).

Thank you for completing our survey!

Key for selection options

1 - I have read and understood the above statements and agree to take part in this survey

Yes

11.a - If yes, how many? ***Please select from drop-down list.***

1
2
3
4
5 or more
Don't know

13.a - If yes, how many? ***Please select from drop-down list.***

1
2
3
4
5 or more
Don't know

15.a - If yes, how many? ***Please select from drop-down list.***

1
2
3
4
5 or more
Don't know

17.a - If yes, how many? ***Please select from drop-down list.***

1
2
3

4
5
6-10
11-20
21-30
31-40
41-50
More than 50
Don't know
